# Leisure as social engagement: does it moderate the association between subjective wellbeing and depression in later life?

**DOI:** 10.3389/fsoc.2023.1185794

**Published:** 2023-08-15

**Authors:** Ashwin Tripathi, Tannistha Samanta

**Affiliations:** ^1^Indian Institute of Technology Gandhinagar, Gandhinagar, India; ^2^Flame University, Pune, Maharashtra, India

**Keywords:** leisure, social engagement, subjective wellbeing, depression, aging, LASI-Wave 1, India

## Abstract

**Objectives:**

To investigate the role of leisure (as social engagement) in moderating the association between subjective wellbeing and depressive symptoms among older Indians.

**Methods:**

The sample included data from 39,538 older adults (aged 55–80) from the Longitudinal Aging Study in India (LASI, Wave-1), 2017–2018. Individual level questionnaire was used to examine the relationship among social engagement, subjective wellbeing, and depressive symptoms. Moderating effects of leisure activities were estimated through interaction analysis and linear multivariable modeling.

**Results:**

Low participation in social engagement activities (or leisure) was associated with greater likelihood of depressive symptoms. Leisure activities positively and significantly moderated the subjective wellbeing among older adults with depressive symptoms. Results suggest a significant wealth gradient where affluent older Indians having a clear advantage in heightened levels of social engagement and subsequently lower likelihood of depressive symptoms. Additionally, being in an urban area, co-residence in a “joint” household and belonging to the dominant social groups in terms of caste and religious categories are associated with gains in wellbeing.

**Discussion:**

The direct and indirect effects of social engagement suggest that depressive symptoms can be mitigated while enhancing overall wellbeing of older adults. This holds promise for social policy in redirecting efforts to develop age-friendly initiatives and social infrastructure that enhance the link between engagement and wellbeing.

## Introduction

The human experience of aging has profound implications for economy, society and policy across the globe. Though sociodemographic trends of increasing life expectancy, declining fertility, changing living arrangements or pension and social security provisioning have been uneven across the world regions, but there is no denying that older adults now, regardless of their geographical location, spend more years in their post-retirement lives. Building on this backdrop, many scholars (Caputo and Simon, 2013; Agarwal et al., 2020) have shown concerns around rising needs for developing long-term care services, ensuring physical and mental wellbeing in later lives. Although the new generations of older adults are more healthy, active and engaged (Katz, 2000; World Health Organization, 2015), as captured by Laslett (1991) seminal concept of the “Third Age” where new forms of engagement and temporal autonomy (Gilleard and Higgs, 2002), that builds on the influential notion of “Successful Aging” (Kahn and Rowe, 1998), is crucial in understanding the continued participation of older people in economic and social activities. However, at the same time, scholars (Djernes, 2006; Goel et al., 2014) have noted the growing instances of geriatric depression among middle-age and older individuals. Psychometric disorders like these, further intensify the complications in gradual increase in physical illness and other risks of new disease and elevates mortality (Hossain et al., 2021). In fact, elderly depression can be considered as the outcome of physical and mental health and further entangled with socio-economic difficulties. Therefore, it becomes crucial to see if notions of successful aging (particularly, activity-based engagement) affect the mental wellbeing of the elderly. As such, there is now a well-established empirical link between “successful” or “productive” aging and overall wellbeing and improved quality of life (Baltes and Smith, 2003; Bowling, 2007). This becomes even more interesting in Asian contexts such as India, where the post-retirement lives are not always constructed around the ideals of “successful” aging; instead, a combination of patriarchal ideologies and economic realities compel different trajectories across gender, class and age groups (Dhal, 2017; Jecker, 2020).

There has been a growing interest in understanding the patterns of time-use among older persons since successful aging has been linked with how time is spent in activities that affect life satisfaction (see Gauthier and Smeeding, 2003, 2010). However, the empirical link between time-use for active and successful aging is not well documented (Kim, 2019; Wanka, 2019; Vilhelmson et al., 2022). Scholars (Sprod et al., 2015, 2017; Strazdins et al., 2016; Weir et al., 2018) have defined how people choose to use their time to engage in various activities of daily life as a prominent indicator of successful aging. This is further delimited by contextual complexities (Samanta, 2018; Lamb, 2020), where few scholars have (Lamb, 2014; Samanta, 2018) critiqued the western ideal of “successful aging” (with its emphasis on the individual self as a project) and argued for alternative cultural models of aging well built around ideals of harmony, self-acceptance and fate-determinism. Nonetheless, irrespective of the cultural context, mental health is an important determinant of successful aging and longevity (Fancourt and Tymoszuk, 2019) but is prone to decline with age due to numerous life events and circumstances experienced by older adults (such as bereavement-led lone living, impoverished social interactions, poor health, retirement) (Fancourt and Tymoszuk, 2019). In fact, to what extent and in what forms current generations of older adults engage in everyday activities remain unknown, especially in the developing world; and how active engagement could provide improved wellbeing remains unaddressed. That said, the role of social support/networks, living with spouse and socio-economic status has been recently explored using the recently released panel data on aging—Longitudinal Aging Survey in India (LASI) baseline survey (Wave 1, 2017–2018) (Hossain et al., 2021; Muhammad and Maurya, 2022; Muhammad and Rashid, 2022) to identify factors that can protect against the development of mental health issues like depression. But the link between social engagement (through leisure) and wellbeing remains underexplored (Adams et al., 2011; Chang et al., 2014). Given this lack, we examine how the everyday social engagement (aka, leisure) activities of older adults are utilized using the LASI dataset. Our exploration of these activities is based on the argument that people's everyday activity patterns—i.e., their time spent on daily activities and for different purpose have crucial implications for individual health and wellbeing (Havighurst, 1968; Weir et al., 2018; Bauman et al., 2019) and more specifically for individuals suffering from depression.

Put simply, the objective of this study was to investigate the role of social engagement through leisure practices in moderating the decreased subjective wellbeing experience among Indian older adults showing depressive symptoms. The study also seeks to understand the impact of socio-demographic features such as social class, urban residence, living arrangements and belonging to dominant social groups (caste and religious categories) on subjective wellbeing. Overall, with the growing aging population and mental health concerns—it is reasonable to examine pathways through which social policies and age-friendly initiatives can be re-directed to develop appropriate age-friendly societies in post-reform India.

### Social-psychological wellbeing in later life: a review of existing scholarship

India is the second most populous country in the world with roughly 138 million^1^ of 60+ persons (~10.1%) in the total population (Arokiasamy et al., 2012). India added 34 million older persons from its last Census (2011) and this number is expected to increase by 56 million by the year 2031 (or 13.1% of the total population). While an alarmist narrative about “population explosion” (Hartmann, 2010) dominated demographic knowledge in the early decades of post-colonial India, this shifted quickly to aging being a “problem” given the changing social (modernization, urbanization, and changing family structures) and policy contexts. It is only recently that an attention to other dimensions of growing old, such as the role of social networks (Hirve et al., 2014; Himanshu et al., 2019), role of affective cultures (Brijnath, 2012; Devi et al., 2021); time-use patterns (Tripathi and Samanta, 2023b) and biometric parameters (Arokiasamy et al., 2012), allow innovative analysis of quality of life among older Indians (Mandi and Bansod, 2022).

One of the emerging public health concerns and a greater social challenge is the prevalence of depression among middle-aged to older people worldwide. India is no exception. For example, in a recent study, Muhammad and Maurya (2022) using LASI data reported that ~35% of the Indian older population suffers from depression. WHO International^2^ (2017) describes depression as a serious mood disorder that can affect the way individuals feel, act and think. It also holds ability to further intensify complications in treating physical illness and one's functionality (Pinquart and Sörensen, 2001; de Jong Gierveld and Hagestad, 2006). Although it is a common psychiatric disorder (Mirowsky and Ross, 2002; Butler, 2004) that can occur in childhood, adolescence, and in later life (Miech and Shanahan, 2000; Hammen, 2009) but impacts far greatly the older population. Chronic depression might also lead to loss of physical health, independence, employment and income, family and friends, house and safe environment (Van Baarsen et al., 2001). Older people across the world have found such event further escalating their health deterioration in case of inadequate coping strategies (Van Baarsen et al., 2001; Blazer, 2003; Drageset, 2004; Batistoni et al., 2007; Shahbazzadeghan et al., 2010; Drageset et al., 2011). Studies from India have consistently shown that those with poor self-rated health and functional limitations are at a significantly higher risk of depressive symptoms. Further, in contexts such as India where patriarchal ideologies restrict older women's access to mobility, re-partnering and economic security, widowhood has been shown to be associated with higher likelihood of late-life depression and cognitive impairment (Srivastava et al., 2021). Additionally, given the familial context of care and instrumental support in older ages, living alone (leading to an erosion of social capital) and residing in a rural area have been shown to be associated with higher proportion of depressive symptomology and feelings of loneliness among older Indians (Samanta, 2014; Muhammad and Meher, 2021).

At the same time, the growing global popularity of the successful aging paradigm, numerous studies have focused on interventions for promoting absence of mental and physical illnesses. Notwithstanding the critics of this paradigm, big cohort studies like *MacArthur Study of Successful Aging* (Berkman et al., 1993) and *Berlin Aging Study* (Baltes and Mayer, 2001) have explored the “what” of successful aging and determined a range of complex physical and cognitive abilities of older men and women functioning at high, medium and impaired ranges and further elaborated on the psychosocial and physiological conditions to distinguish among the old age groups. Thus, segregating the young-old from the old-old cohorts which are further influenced by the contemporary changes of consumerism and globalization. Escolar Chua and De Guzman (2014) experimental study on the effects of community-based third age learning program among older Filipino, showed that the treatment group showed higher life satisfaction, increased self-esteem and lower levels of depression. Similarly, numerous other studies have linked (social) engagement activities of older adults to positive health related outcomes (Aldridge and Lavender, 2000; Dench and Regan, 2000; Swindell, 2002; Elderhostel Inc., 2007; Formosa, 2010).

### Third age, social engagement, and wellbeing: examining the pathways

Given the recent interest in gerontological scholarship on how late adulthood is lived, scholars have examined the link between Third Age lifestyles and overall wellbeing in later life. To be sure, Third Age is recognized as a (post-retirement) time for personal fulfillment and vitality among those who have acquired adequate savings and are hence able to make lifestyle-based consumerist choices. For example, research from Poland (Zadworna, 2020) showed that retirees who are engaged in Third Age related interventions (e.g., educational activities that promote lifelong learning) reported higher levels of self-rated health and life satisfaction than those who were not participating in such activities. Again, earlier studies have shown how Third Age activities such as volunteering and other forms of civic engagement serve as a vehicle through which social capital is reaped. This in turn promotes a heightened sense of self-worth and wellbeing among older adults (Hendricks and Cutler, 2004). Their active engagement in diverse forms of activities contributes toward their mental and physical alertness. There exists ample evidence for this positive correlation that can reduce the burden of health expenditures associated with population aging (Krawczynski and Olszewski, 2000; Cohen, 2005; Elderhostel Inc., 2007; Flood and Phillips, 2007; Nadasen, 2007; Hanna and Perlstein, 2008) and cognitively stimulating activities through sustained commitment to lifelong learning has led to increased life satisfaction and hence, successful (active/productive) aging (Escolar Chua and De Guzman's, 2014). Following Arai and Pedlar (2003), who argue toward emphasizing community, social capital and communal leisure practices to explore the social relevance of leisure, we explore the social engagement activities from LASI survey. As such, the activity theory (Havighurst, 1961; Longino et al., 1982) in gerontology which is often used as a shorthand for explaining increased wellbeing and reduced social isolation among older persons is built on the premise that (social) activity through satisfaction with outcomes, mental stimulation and personal routines improve self-efficacy. While health benefits of physical activity are well-established in gerontological tradition (Lancet, 2021), researchers have increasingly recognized the importance of social support pathways with friends, family kinship ties as promoting cognitive reappraisals making stressful situations less stressful (Lazarus and Folkman, 1984; cited in Adams et al., 2011). Building on this body of work, the current paper conceptualizes leisure as social engagement. The LASI survey offers a useful empirical site to examine the association between social engagement-based leisure activities and wellbeing in later life.

**Figure 1:**
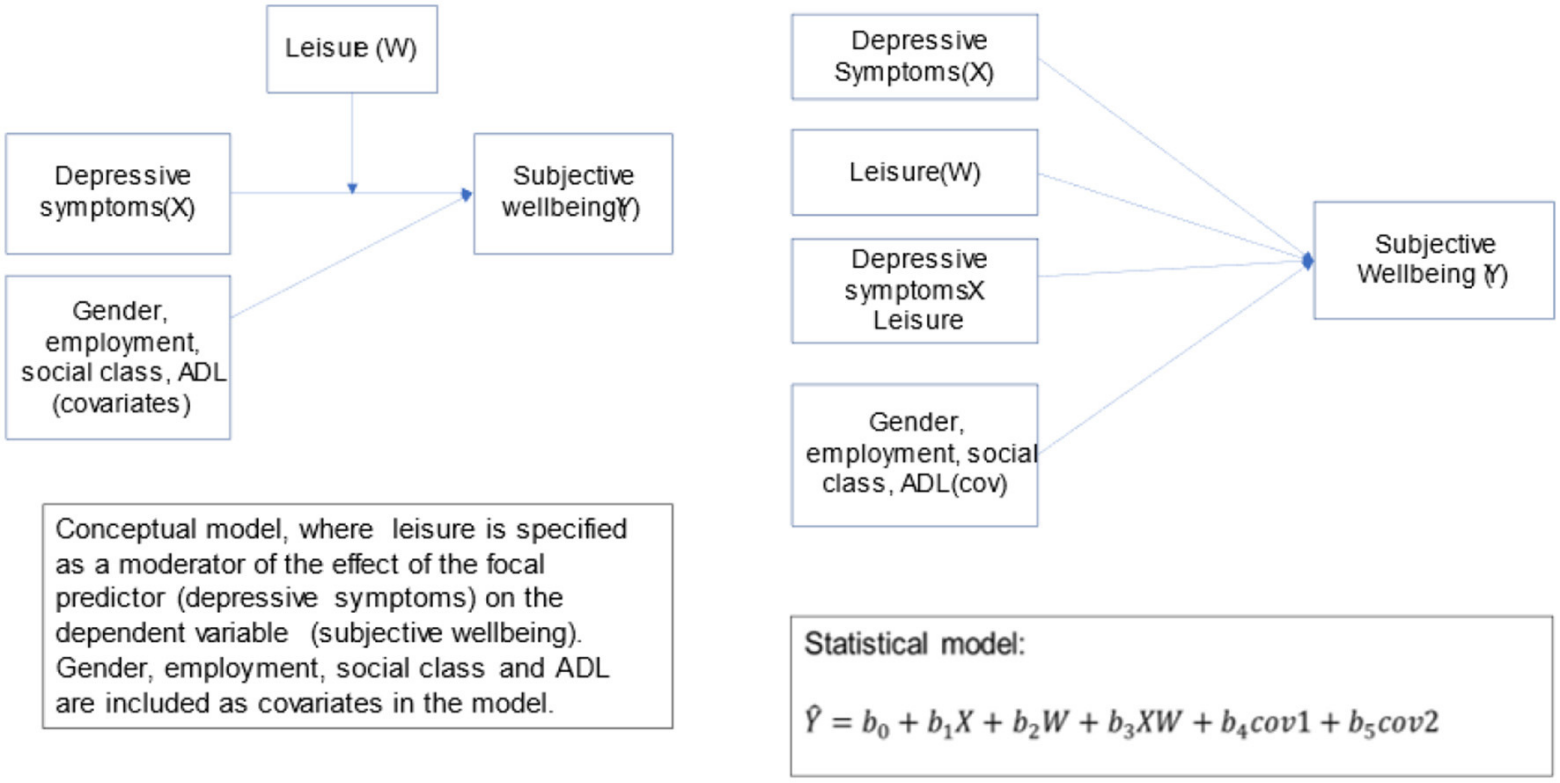
Conceptual model.

The activity-wellbeing link has been a key signifies for the Third Age lifestyle globally. In India, Third Agers are primarily urban, (upper) middle-class older adults—a growing cohort of “new-age elderly” (Samanta, 2018; Lamb, 2020)—who have been participants of outdoor leisure/travel, housing projects, fashion and lifestyle-based markets. This shift has ushered a post-retirement life among urban (upper) middle-class older Indians where leisure and experience can be “purchased without a loss of a productive and vital self” (Samanta, 2018, p. 95). Replacing the traditionally aspired dependence discourse of old age-commonly associated with aging in family-centric contexts-with leisure-based consumptive lifestyles, have motivated cultural anthropologists to remind us how the access and availability of free time varies across the social class (Samanta, 2018; Lamb, 2020). As such, this story about the politically influential middle to upper middle-class Indians with adequate residual spending power and cultural capital to engage in age-ambiguous consumer citizenry, is now well documented by academic commentators in India (Fernandes and Heller, 2006; Srivastava, 2007; Baviskar and Ray, 2020). In our previous work, we operationalize third age in the context through active participation in social engagement activities, as authors have noted that time-use is a valuable descriptor of people's lifestyle, as studying how people spend their time is critical for understanding the determinants and consequences of individuals' overall wellbeing and life satisfaction (Tripathi and Samanta, 2023a,b). On similar lines, Padhy et al. (2015) explores the positive relationship between leisure activities, wellbeing and life-satisfaction among young adults through leisure motivation. However, for a long time India has struggled to collect data on time-use variabilities of gender and generation (Hirway, 2021). Only recently, we have two nationally representative datasets—(i) LASI data which utilizes the stylized questions method in elucidating the time-use of older adults in their experimental module and (ii) the National Sample Survey Organization (NSSO) (2017–2018) that uses the time-diary approach in collecting time-use information. As we look into the completely unexplored area of older adults' time allocation pattern, we explore the LASI data to study the impact of social engagement activities with subjective wellbeing of the Indian elderlies. For this, we hypothesize the following below. Figure 1 shares the conceptual model for this study.

### Hypothesis

H_1_: Older adults with higher depressive symptoms are more likely to report lower levels of SWB, regardless of their socio-demographic characteristics.

H_2_: Heightened levels of leisure (here, social engagement) are associated with advantageous socio-economic characteristics such as economic affluence, living with child(ren)/spouse and social affiliation with dominant castes and religious categories. As a corollary to this hypothesis, we argue that the leisure-socioeconomic nexus is a determinant of depressive symptoms and overall SWB.

## Methods

### Data and sample

#### LASI data set

This study utilized data from the nationally representative survey of the LASI Wave 1 (2017–2018), which investigates the health, economic, and social determinants and consequences of population aging in India. The representative sample included 72,250 individuals aged 45 years and above and their spouses across all states and union territories (UTs) of India except Sikkim. LASI adopts a multistage-stratified area probability cluster sampling design to select the eventual units of observation (a three-stage sampling design in rural areas and a four-stage sampling design in urban areas) (Detailed information on the sampling frame is available in https://www.iipsindia.ac.in/sites/default/files/LASI_India_Report_2020_compressed.pdf). The LASI survey provides scientific evidence on demographics, household economic status, chronic health conditions, symptom-based health conditions, functional and mental health, biomarkers, healthcare utilization, work and employment etc. Further, an individual survey schedule was administered to each consenting respondent aged 45 and above and their spouses (irrespective of age) in the sampled households. In addition, the LASI includes an individual module on biomarkers and direct health examination. The present study is based on the eligible respondents who are aged 55–80 years. The effective sample size for the present study was 39,538 older adults aged 55 and above, including 18,687 males and 20,851 females.

## Measures

### Depressive symptoms

In LASI, two internationally validated and comparable tools are used to assess depressive symptoms and episodes: the Center for Epidemiologic Studies Depression (CES-D) scale was used to identify the presence of depressive symptoms (Radloff, 1977) and the Composite International Diagnostic Interview—Short Form (CIDI-SF) scale, a structured interview scale, was used for diagnosing probable major depression (Kessler and Üstün, 2004). The CESD comprised of 10-item scale including questions (a) *How often did you have trouble concentrating*?, (b) *How often did you feel depressed?*, (c) *How often did you feel tired or low in energy?*, (d) *How often were you afraid of something?*, (e) *How often did you feel you were overall satisfied?*, (f) *How often did you feel alone?*, (g) *How often were you bothered by things that don't usually bother you?*, (h) *How often did you feel that everything you did was an effort?*, (i) *How often did you feel hopeful about the future?*, and (j) *How often did you feel happy?*. The Cronbach score for depressive symptoms was 0.79. Negatively framed questions were reversed scored ranging from (1) rarely or never (<1 day) to (4) Most or all the time (5–7 days). The scores were summed, with higher scores indicating higher levels of depressive symptoms (range 4–40). A depression dummy variable was created with scores <17 being coded as 0 and above 17 coded as 1. As the CESD scale for depression monitoring is globally acceptable and a score of more than 16 is considered to be depressed, we stick to the score 17 for creating our dummy variable (Muhammad et al., 2022).

### Subjective wellbeing

In this paper, we use the LASI life satisfaction inventory that was assessed with a five-item scale that asks people how satisfied they are with their life (a) *In most ways my life is close to ideal*, (b) *the conditions of my life are excellent*, (c) *So far, I have got the important things I want in life*, (d) *If I could live my life again, I would change almost nothing*. In the questionnaire, the respondents were provided with seven response categories, ranging from (1) Strongly disagree to (7) Strongly Agree. A higher value indicating higher LS (continuous). This is a standard measure-defined as the self-assessed worth of an individual's life- used in other health surveys and reflects overall wellbeing of respondents in a reliable way (see Stephen et al., 2014 for a discussion on the age-related changes of multiple wellbeing measures).

This was collected under the family and social network section of the LASI (2017–2018) questionnaire that also included questions related to discrimination (ill treatment) and psychosocial measures (life satisfaction, religiosity). We use psychosocial measures as a proxy for older adults' subjective wellbeing. The Cronbach score for subjective wellbeing is 0.89.

### Leisure as social engagement

Defined as the extent to which an individual engages in social activities that are meaningful to them (Kumar et al., 2006), social activities were measured in LASI according to the reported frequency of participation in 11 social activities (Cronbach alpha 0.65). We use the social engagement as a proxy for their leisure. Examples include “[Attending] an educational or training course,” and “[Going] to a sport, social, or other club”. Frequency of participation in each activity was reported on a 7- point Likert scale ranging from 1 = “daily” to 7 = “never”. This was recoded into binary categorical variable −1 = “yes” and 0 = “no”. We created an additive summary index of the social activities' variables, where higher scores were indicative of higher social activities (range: 0–13). For analysis purposes, we created dummy binary variable (0 for no leisure activities and 1 for leisure activities ranging from 1 to 13).

### Covariates

Other variables considered in analyses include age (years), gender, wealth index (proxy for social class) number of limitations of daily living (ADL; range: 0–3+), living arrangements, education and caste are utilized in analyzing the role of leisure on SWB among older adults with depressive symptoms.

### Analysis

Sample distributions of demographic and health characteristics were compared across major depression diagnostic groups using *t*-tests and chi-square tests (α = 0.05). This was followed by a Spearman's rank correlation analysis to identify potential sociodemographic, health, and behavioral predictors of depressive symptoms and subjective wellbeing. For instance, spearman correlation for depression and subjective wellbeing is −0.046, subjective wellbeing and leisure is 0.0784 and between leisure and depression is 0.032 (all at *p* < 0.001). Once we got the correlation coefficients, we identified potential variables across which the empirical model could be built. For the final analysis, we fit (two models) independent models for each measure of depression (CESD scale values), life satisfaction, and leisure. In each model, we estimated the total effect of depression on subjective wellbeing of older adults and the effect of social engagement via leisure/social engagement activities. The assumption of normality of the outcome/dependent variable was analyzed through skewness and kurtosis in the descriptive analysis. Similarly, homoscedasticity and absence of multicollinearity was confirmed through scatterplot and crosstabs, respectively. We first fit an unadjusted bivariate linear regression model estimating the association between depression and subjective wellbeing. We also added potential confounders to the model. These analyses were performed using Stata software (version 16).

### Regression model


(1) $$\begin{array}{l} y_{\text{i}}={\text{β}}_{0}+{\text{β}}_{1}x_{\text{i}}+{\text{β}}_{1}z_{\text{i}}+{\text{ε}}_{\text{i}} \end{array}$$

In the above Equation (1), y represents the predicted/outcome variable, i.e., subjective wellbeing of older adults age 55–80 years old. This is understood as the function of (i) depressive symptoms, depicted by x and (ii) social engagement activities shown by z. While β_0_ represent the intercept, i.e., the constant value of subjective value when both x (depressive symptoms) and z (leisure) is equal to zero. At last, ε_i_ represents the residual/unexplained variation in the model that has not been taken into consideration. Below are more detailed equations to explain our theoretical models:


(2) $$\begin{array}{lll} {\text{SWB}}_{\text{i}}\left(\text{y}\right) & = & {\text{β}}_{0}+{\text{β}}_{1}{\text{Depression}}_{\text{i}}+{\text{β}}_{2}{\text{Leisure}}_{\text{i}}+{\text{ε}}_{\text{i}} \end{array}$$

(3) $$\begin{array}{l} {\text{SWB}}_{\text{i}}\text{(y) }={\text{ β}}_{0}{\text{ + β}}_{1}{\text{Depression}}_{\text{i}}{\text{ + β}}_{2}{\text{Leisure}}_{\text{i}}\\ \;+{\text{ β}}_{3}{\text{(Depression*Leisure)}}_{\text{i}}{\text{ + ε}}_{\text{i}} \end{array}$$

As per Equations (2) and (3), we now run these models in STATA-16 by also incorporating the covariates (discussed above) including gender, employment status, wealth index, living arrangements, education, caste, religion and ADL limitations in our sample to study the relationship.

## Results

Descriptive statistics are detailed in Table 1. We considered older adults 55–80 years old. Age groups 55–60 and 61–65 comprise nearly 50% of our analytical sample. In both the age groups, we have more women respondent than men. The *t*-statistic test does show significant differences between both the genders across education, residence and wealth index. This is not presented in the tables but *t*-test was run in the background. Only 11% of our sample is aged 75 and above. This is expected as with age, the population size reduces and older adults with relatively better health have more chances of surviving. The data has been collected from urban and rural India but most female older adults show (~67%) show no education. This statistic is better among the men, where only ~35% men have had no access to education. Most of the study participant belonged to rural areas (66% men and 65% women). In terms of class structure, we operationalize social class using the wealth index that was collected as part of monthly per capita expenditure of older individuals. ADL limitations are quite common among older adults that includes concerns regarding struggles in doing everyday activities. Again, women show more limitations that men, where ~50% of the women population have 1–3 limitations while only ~32% of men show similar concerns. Intergenerational residence (living with spouse and is the most dominant form of living arrangement in the LASI sample (~90% either living with their spouse/children). This is consistent with other studies that show the centrality of “joint family” as the defining characteristic of Indian households. Finally, we included variables characterizing social groupings in terms of caste (where “lower” caste groups include Scheduled Tribes, Scheduled Castes and Other Backward Castes, while the “forward” caste includes those in the “general” category). We know for a fact that Indian society remains highly stratified with status distinctions and hierarchies established through the routes of marriage, occupations, diet and lifestyle (see Desai, 2011; Natrajan and Jacob, 2018).

**Table 1:** Socio-demographic characteristics of LASI (2017-18) sample.

**Socio-demographic features**	**Total (*N* = 39,538)**	**Men (*N* = 18,687)**	**Women (*N* = 20,851)**
**Age group**			
55–59	10,167 (25.71)	4,560 (24.40)	5,607 (26.89)
60–64	10,267 (25.97)	4,707 (25.19)	5,560 (26.67)
65–69	8,944 (22.62)	4,381 (23.44)	4,563 (21.88)
70–74	5,811 (14.70)	2,933 (15.70)	2,878 (13.80)
75–80	4,349 (11.00)	2,106 (11.27)	2,243 (10.76)
**Education**			
No education	20,235 (51.18)	6,419 (34.35)	13,816 (66.26)
Primary	9,689 (24.51)	5,549 (29.70)	4,140 (19.86)
Secondary	6,390 (16.16)	4,336 (23.20)	2,054 (9.85)
Higher	3,223 (8.15)	2,382 (12.75)	841 (4.03)
**Residence**			
Rural	26,006 (65.77)	12,407 (66.39)	13,599 (65.22)
Urban	13,532 (34.23)	6,280 (33.61)	7,252 (34.78)
**Wealth index**			
Poor	16,189 (40.95)	15,396 (38.94)	8,707 (41.76)
Middle	7,953 (20.11)	3,770 (20.17)	4,183 (20.06)
Affluent	15,396 (38.94)	7,435 (39.79)	7,961 (38.18)
**Caste categories**			
OBC	6,596 (16.97)	3,105 (16.87)	3,491 (17.06)
SC/ST	6,509 (16.74)	3,047 (16.55)	3,462 (16.92)
Upper caste (unreserved category)	14,865 (38.24)	7,116 (38.66)	7,749 (37.87)
**Religion**			
Hindu	28,503 (73.33)	13,544 (73.58)	14,959 (73.10)
Muslim	4,554 (11.72)	2,119 (11.51)	2,435 (11.90)
Christian	3,808 (9.80)	1,775 (9.64)	2,033 (9.93)
Others	2,007 (5.16)	970 (5.27)	1,037 (5.07)
**ADL Limitations**			
0	23,002 (58.18)	12,531 (67.06)	10,471 (50.22)
1	4,381 (11.08)	2,010 (10.76)	2,371 (11.37)
2	2,992 (7.57)	1,163 (6.22)	1,829 (8.77)
3+	9,163 (23.18)	2,983 (15.96)	6,180 (29.64)

Source: LASI Wave 1 (2017–18), Individual questionnaire, *n* = 39,538; weighted; (%s in parentheses).

Table 2 shows the mean values and percentage of older adults for our three variables of interest. First, subjective wellbeing of older men and women does differ significantly and have a mean score of 24.22 for men and slightly lower for women at 23.70. Similarly, 46.9% men show depressive symptoms, while 49.6% women show similar effects. Again, more men engage in leisure activities (83.6%) while substantially lower women (79.3%) engage in leisure activities. We discuss the items included in each of our variables of interests in Table 2 in the methods section above.

**Table 2:** Descriptive statistics for variables of interest.

**Variable**		**Men**		**Women**		**Pairwise *t*-test**
	***N***	**Mean/(SE)**	***N***	**Mean/(SE)**	***N***	**Mean difference**
Subjective wellbeing (SWB)	18,118	24.221	20,359	23.705	38,477	0.516^***^
		−0.053		−0.051		
Depression	18,111	17.692	20,363	18.032	38,474	−0.340^***^
		−0.038		−0.037		
Leisure	18,686	2.099	20,851	2.019	39,537	0.080^***^
		−0.003		−0.003		

Source: LASI Wave 1 (2017–18), Individual questionnaire; weighted. Statistical significance at ^***^p < 0.01.

In Table 3, represents nested, multivariate linear regression as also shown by Equation (2). We see for older adults who have depressive symptoms (continuous variable) show decreased subjective wellbeing (β_1_ = −0.186^***^). More specifically, if an older individual experience depressive symptom(s), the subjective wellbeing of individuals age 55–80 years old decrease by 18.6%. While for older adults who show no depressive symptoms, have a positive association with subjective well–being (β_0_ = 23.45^***^). With one unit increase in social engagement activities (as leisure) subjective wellbeing increases by ~91%. Both the above stated results are statistically significant. Our theoretical motivation of older adults' social participation in improving overall/subjective wellbeing was further explored by interacting our two explanatory variables (depressive symptoms and social engagement activities). By interacting them (as shown in Equation 3), we see that individuals who show depressive symptoms but also engage in social activities, have positive impact on depressive symptoms (β = 0.0522^***^). Thus, positively impacts their subjective wellbeing and is statistically significant as well.

**Table 3:** Multivariate linear regression estimates for depression and subjective wellbeing as moderated by leisure activities.

**Variables**	**Subjective well being (independent)**
Leisure	0.913^***^
	(0.307)
Depression	−0.186^***^
	(0.0333)
Depression ^*^ Leisure	0.0522^***^
	(0.0161)
Constant	23.45^***^
	(0.634)
Observations	38,418
*R* ^2^	0.015

Robust standard errors in parentheses.

^***^p < 0.01.

^**^p < 0.05.

^*^p < 0.1.

We also see how this association varies across socio-demographic characteristics such as age, social class, living arrangements, caste and education. There is a clear age gradient in improved subjective wellbeing vis-à-vis leisure participation. For instance, as individuals age, their subjective wellbeing enhances, though the effect is not very neat. To complement this finding, we also see those individuals with activities of daily (ADL) limitations experience 49% less subjective wellbeing when compared to groups having no activities of daily limitations. Similarly, people living with spouse or children and with spouse and children (i.e., living in joint family structures) experience more subjective wellbeing than living alone or with others (β = 2.4^***^). There also exists a strong caste component in the Indian context (check Appendix Table A1) where material and non-material gains (or otherwise) caste and social class belonging go hand in hand. For example, in Appendix Table A1, we see that older adults from unreserved caste experience 1.36% more subjective wellbeing compared to their reserved counterparts.

## Discussion

### The role of social engagement activities through leisure in enhancing the subjective wellbeing of older individuals with depressive symptoms

This paper examines the moderating role of social engagement activities among older individuals with depressive symptoms. This paper builds on the existing scholarship on Third Age that shows heightened levels of social engagement and leisure pursuits being associated with gains in overall later life wellbeing. We mined the newly released Longitudinal Aging Study in India (LASI, 2017-18) data to show how social engagement activities, specifically leisure-based activities such as eating outside, playing, visiting friends and relatives and many more, to improve the subjective wellbeing of the older adults, even after controlling for their gender, age, social class, employment status and ADL limitations. As hypothesized, subjective wellbeing decreases with increased depressive symptoms. Previous studies, as described in our literature review, have emphasized this relationship between mental health issues and overall wellbeing of older individuals. As the social context of aging among the urban middle class in India is fast changing, we attempted to critique the alarmist narrative of social disengagement, dependence and debility that are commonly associated with later life. In fact, as suggested earlier, leisure-based activities and social engagement have remained outside the scope of gerontological scholarship in India. Some recent studies based on the baseline LASI data (which is currently cross-sectional in nature) focus on health inequalities depression and cognitive functioning, elder abuse and depression (Muhammad et al., 2022), physical limitations and depressive symptoms (Hossain et al., 2021).

We hypothesized a negative association between depression and subjective wellbeing being moderated through social engagement as leisure (H_1_). That is, engagement in leisure activities improves subjective wellbeing of individuals and more so among older adults with reported depressive symptoms. Our results reveal a positive influence of social engagement activities in mitigating the effects of depression among the Indian older adults and improving their subjective wellbeing (H_2_) (as presented in Table 3). Our broader motivation for this paper lies at the emergence of Third Age (as a predictor for “successful aging”) and how it relates to improved subjective wellbeing (Fancourt and Tymoszuk, 2019). In the process, we have shown that with older adults with no physical limitations experience improved wellbeing. Again, similar to other studies on older adult health outcomes (Gupta and Coffey, 2020; Vyas et al., 2022), we notice social group differentials in subjective wellbeing; specifically, being in the dominant caste (forward/none) and belonging to the affluent social class are associated with gains in subjective wellbeing. All in all, our analyses point to the Third Age lifestyle as an important predictor for aging successfully among urban middle-class older Indians.

### Concluding remarks

Mental health is prone to decline with age because of life events (and thus increase in depressive symptoms) and circumstances commonly experienced by older adults. Many older individuals experience lone living, impoverished social interactions, poor health, retirement and worsening economic conditions (Barrientos et al., 2003). Many a times, it remains underdiagnosed and under treated that further leads to higher risk of dementia and other health shocks (Gupta and Coffey, 2020). Much research has been undertaken to identify factors that can protect again the development of depression, including social networks and social support, physical activity and cognitive stimulation. However, our study does not argue for preventive actions rather emphasizes the importance of social activities in mitigating the effects of depression on the subjective wellbeing of Indian older adults.

A similar study by Fancourt and Tymoszuk (2019) on their work on English Longitudinal Study of Aging (ELSA) argue that there has been growing research in demonstrating the effects of cultural engagement on depression. This includes studies of both active and receptive cultural engagement. Most of these studies, including ours have centered around the impact of cultural engagement on mitigating the effects of depression on the overall wellbeing of individuals. But we also argue that social engagement activities can also reduce the risk of depression in later years. This remains relevant, more so in the Indian context, as discussion around mental health among older individuals remain a tabooed topic. This could have led to non-response biases in the data among participants who experienced depressive episodes but did not report it.

One of the strengths of our study is the nationally representative older adults' baseline dataset which would incorporate subsequent waves of data collection in the coming years. Also, LASI uses well-validated measure of depression (CES-D scores) and has tested different thresholds (as the scale has been developed in this study through additive scores).

### Limitations

Our study is however not without limitations. One of the empirical challenges we faced is the possibility of reverse causality of our interest variables. It is unclear if people who report high levels of subjective wellbeing (and lower likelihood of depression) are also the ones who engage in heightened levels of social activities. This raises the issue of reverse causality in the analysis of the relationship between subjective wellbeing, depression and leisure engagement activities. More specifically, this suggests that it is uncertain whether individuals who report higher levels of subjective wellbeing and lower likelihood of depression are the ones who actively engage in more social activities. We tried to partially mitigate the above problem by running our models with and without individuals with depression (results not reported in the paper) and we received similar results. Moreover, the LASI data does not provide data for individuals who are undergoing psychiatric treatment or medications (i.e., medically diagnosed cases of mental health issues). In addition to medically appropriate ethical data collection efforts, longitudinal/panel studies will help address questions of causality and selection bias thereby offering directions for effective and targeted interventions. Also, the findings also suggest the need to use qualitative assessment that explored the role of additional factors have on population's subjective wellbeing, thereby shaping public policy and form the foundation of intervention programs.

### Implications

The paper highlights the importance of leisure activities in the lives of older adults. Through empirical evidence, we show that low participation in social engagement activities is associated with a higher likelihood of experiencing depressive symptoms. Therefore, we argue the importance of promoting and encouraging social engagement activities among older adults to enhance their overall subjective wellbeing. Interventions at policy level needs to consider aspects other than health in engaging older adults in their later lives. More specifically, create age-friendly initiatives and infrastructure that encourages engagement and improve mental health outcomes. Moreover, our findings also suggest a strong wealth gradient, where affluent older Indians experience heightened sense of social engagement. Therefore, policies need to address the inequities in accessing leisure, irrespective of their socio-economic status. Similarly, addressing the urban-rural differences in providing better access to social engagement opportunities, infrastructure and resources to contribute toward better wellbeing outcomes. Future research and interventions need to consider socio-cultural factors in planning initiatives, interventions and programs to provide more culturally sensitive and tailored needs in the context of older adults in India.

## Data availability statement

Publicly available datasets were analyzed in this study. This data can be found here: https://www.iipsindia.ac.in/content/LASI-data.

## Author contributions

All authors listed have made a substantial, direct, and intellectual contribution to the work and approved it for publication.

## Footnotes

^1^ https://www.mospi.gov.in/documents/213904/301563/Elderly%20in%20India%2020211627985144626.pdf/a4647f03-bca1-1ae2-6c0f-9fc459dad64c

^2^Mental health of older adults (who.int) (https://www.who.int/news-room/fact-sheets/detail/mental-health-of-older-adults) (accessed March 10, 8:25 a.m.).

## Appendix

**Table A1:** Nested multivariate linear regression analysis for subjective wellbeing, leisure, and socio-demographic variables, predicting depressive symptoms (*N* = 38,386, LASI 2017–18).

**Variables**	**SWB (Co-efficient/ SE)**	**SWB (Co-efficient/ SE)**
Leisure	0.857^***^	0.394
	(0.308)	(0.307)
Depression	−0.187^***^	−0.149^***^
	(0.0333)	(0.0332)
Leisure * Depression	0.0535^***^	0.0450^***^
	(0.0161)	(0.0160)
Age 60–64 (Ref: 55–59)	0.0599	0.184*
	(0.101)	(0.102)
Age 65–69	0.163	0.492^***^
	(0.105)	(0.108)
Age 70–74	0.0235	0.516^***^
	(0.120)	(0.126)
Age 75–80	0.133	0.779^***^
	(0.133)	(0.142)
Gender (= Female)	−0.332^***^	0.138*
	(0.0736)	(0.0828)
1 ADL Limitations (Ref: No ADL Limitations)		−0.548^***^
		(0.119)
2 ADL Limitations		−0.911^***^
		(0.141)
3 ADL Limitations		−1.676^***^
		(0.0961)
Living with Spouse (Ref: Living alone)		263***
		(0.195)
Living with Spouse and Children		2.920^***^
		(0.186)
Living with Children and others		2.285^***^
		(0.191)
Living with others only		1.111^***^
		(0.246)
Working (Ref: Unemployed/retired)		−0.185**
		(0.0841)
Middle class (Ref: Poor)		0.425^***^
		(0.0996)
Affluent class		0.761^***^
		(0.0852)
Muslim (Ref: Hindu)		−0.603^***^
		(0.118)
Christian		0.496^***^
		(0.132)
Others		0.965^***^
		(0.168)
OBC (Ref: SC/ST – Lower caste)		0.577^***^
		(0.0916)
Other (Unreserved/Upper Caste)		1.369^***^
		(0.103)
Constant	23.65^***^	20.87^***^
	(0.640)	(0.672)
Observations	38,418	37,765
R-squared	0.016	0.042
